# Supplementary material for: Mechanistic insights into triclosan-induced hepatotoxicity: A network toxicology and molecular docking approach
Source: PLoS One. 2026 Feb 25;21(2):e0333244. doi: 10.1371/journal.pone.0333244 (PMC12935200; doi:10.1371/journal.pone.0333244)
Supplement: S1 Table — (DOC) [file pone.0333244.s001.doc]

S1 Table. The top 10 Gene Ontology terms of 683 potential target genes

| **GOterm** | **subgroup** | **Count** |
| --- | --- | --- |
| response to xenobiotic stimulus | Biological process | 69.00 |
| positive regulation of gene expression | Biological process | 89.00 |
| positive regulation of transcription by RNA polymerase II | Biological process | 110.00 |
| response to hypoxia | Biological process | 43.00 |
| response to nutrient | Biological process | 26.00 |
| response to ethanol | Biological process | 33.00 |
| positive regulation of miRNA transcription | Biological process | 24.00 |
| positive regulation of cell population proliferation | Biological process | 60.00 |
| xenobiotic metabolic process | Biological process | 31.00 |
| negative regulation of apoptotic process | Biological process | 58.00 |
| extracellular exosome | Cellular component | 187.00 |
| extracellular space | Cellular component | 156.00 |
| extracellular region | Cellular component | 157.00 |
| cytosol | Cellular component | 268.00 |
| mitochondrion | Cellular component | 121.00 |
| collagen-containing extracellular matrix | Cellular component | 53.00 |
| protein-containing complex | Cellular component | 66.00 |
| cell surface | Cellular component | 61.00 |
| mitochondrial matrix | Cellular component | 49.00 |
| platelet alpha granule lumen | Cellular component | 20.00 |
| identical protein binding | Molecular function | 159.00 |
| protein homodimerization activity | Molecular function | 88.00 |
| enzyme binding | Molecular function | 58.00 |
| protein binding | Molecular function | 497.00 |
| oxidoreductase activity | Molecular function | 40.00 |
| growth factor activity | Molecular function | 32.00 |
| protease binding | Molecular function | 27.00 |
| heme binding | Molecular function | 29.00 |
| flavin adenine dinucleotide binding | Molecular function | 19.00 |
| integrin binding | Molecular function | 27.00 |
