# Supplementary material for: Mechanistic insights into triclosan-induced hepatotoxicity: A network toxicology and molecular docking approach
Source: PLoS One. 2026 Feb 25;21(2):e0333244. doi: 10.1371/journal.pone.0333244 (PMC12935200; doi:10.1371/journal.pone.0333244)
Supplement: S2 Table — (DOC) [file pone.0333244.s002.doc]

S2 Table. The top 20 pathways of 683 potential target genes

| **term** | **ratio** | **pvalue** | **count** |
| --- | --- | --- | --- |
| Pathways in cancer | 16.7844523 | 1.14E-21 | 95 |
| AGE-RAGE signaling pathway in diabetic complications | 6.890459364 | 8.03E-21 | 39 |
| Lipid and atherosclerosis | 9.717314488 | 1.35E-19 | 55 |
| PI3K-Akt signaling pathway | 12.01413428 | 1.16E-16 | 68 |
| Non-alcoholic fatty liver disease | 7.4204947 | 8.62E-16 | 42 |
| Bile secretion | 5.477031802 | 5.63E-15 | 31 |
| HIF-1 signaling pathway | 6.007067138 | 7.94E-15 | 34 |
| Fluid shear stress and atherosclerosis | 6.713780919 | 2.05E-14 | 38 |
| Alcoholic liver disease | 6.713780919 | 4.22E-14 | 38 |
| Carbon metabolism | 6.007067138 | 4.40E-14 | 34 |
| Metabolic pathways | 29.32862191 | 1.67E-13 | 166 |
| Apoptosis | 6.360424028 | 2.00E-13 | 36 |
| Glycolysis / Gluconeogenesis | 4.416961131 | 5.13E-13 | 25 |
| Endocrine resistance | 5.300353357 | 6.96E-13 | 30 |
| TNF signaling pathway | 5.653710247 | 3.49E-12 | 32 |
| Colorectal cancer | 4.770318021 | 6.65E-12 | 27 |
| IL-17 signaling pathway | 4.946996466 | 9.70E-12 | 28 |
| Hepatitis B | 6.360424028 | 5.49E-11 | 36 |
| Chemical carcinogenesis - receptor activation | 7.4204947 | 6.36E-11 | 42 |
| Adipocytokine signaling pathway | 4.06360424 | 9.49E-11 | 23 |
