# Supplementary material for: Mechanistic insights into triclosan-induced hepatotoxicity: A network toxicology and molecular docking approach
Source: PLoS One. 2026 Feb 25;21(2):e0333244. doi: 10.1371/journal.pone.0333244 (PMC12935200; doi:10.1371/journal.pone.0333244)
Supplement: S3 Table — (DOC) [file pone.0333244.s003.doc]

S3 Table. Results of core genes obtained by three different algorithms in Cytoscape

| **Methods** | **Genes** |
| --- | --- |
| CytoNCA | IL1B/CYP1A1/IGF1/CYP3A4/RELA/MYC/BCL2/SRC/TP53/CASP3/HSP90AA1/EGFR/PRKACA/AKT1/JUN/IL6/ESR1/PPARG/FN1/TNF |
| MCODE | IL4/CXCL2/CSF2/IL13/CXCL1/RELA/CXCL8/JUN/IL1A/IL1B/TNF/IL6/CCL2 |
| CytoHubba | CXCL8/CCL2/IL6/TNF/IL1B/CXCL1/IL1A/CYP1A1/CYP3A4/CYP1A2 |
