# Supplementary material for: Mechanistic insights into triclosan-induced hepatotoxicity: A network toxicology and molecular docking approach
Source: PLoS One. 2026 Feb 25;21(2):e0333244. doi: 10.1371/journal.pone.0333244 (PMC12935200; doi:10.1371/journal.pone.0333244)
Supplement: S4 Table — (DOC) [file pone.0333244.s004.doc]

S4 Table. Sankey diagram dataset of the top 20 pathways for the core targets

| **Description** | **GeneRatio** | **pvalue** | **geneID** | **Count** |
| --- | --- | --- | --- | --- |
| Lipid and atherosclerosis | 0.586206897 | 3.72344E-19 | JUN/HSP90AA1/CXCL8/SRC/CXCL1/CXCL2/TNF/RELA/IL6/IL1B/CASP3/CYP1A1/BCL2/CCL2/AKT1/PPARG/TP53 | 17 |
| IL-17 signaling pathway | 0.482758621 | 5.57667E-19 | JUN/HSP90AA1/CXCL8/CSF2/IL13/CXCL1/CXCL2/TNF/RELA/IL4/IL6/IL1B/CASP3/CCL2 | 14 |
| AGE-RAGE signaling pathway in diabetic complications | 0.413793103 | 6.65703E-15 | IL1A/IL6/JUN/CXCL8/IL1B/CASP3/BCL2/FN1/CCL2/AKT1/TNF/RELA | 12 |
| Pathways in cancer | 0.620689655 | 2.9482E-14 | JUN/HSP90AA1/CXCL8/IL13/FN1/IGF1/ESR1/EGFR/RELA/IL4/IL6/MYC/CASP3/BCL2/AKT1/PPARG/PRKACA/TP53 | 18 |
| Amoebiasis | 0.379310345 | 4.60673E-13 | IL6/CXCL8/CSF2/IL1B/CASP3/FN1/CXCL1/PRKACA/TNF/CXCL2/RELA | 11 |
| Chemical carcinogenesis - receptor activation | 0.448275862 | 1.02739E-12 | JUN/HSP90AA1/SRC/CYP3A4/ESR1/EGFR/RELA/MYC/CYP1A2/CYP1A1/BCL2/AKT1/PRKACA | 13 |
| Human cytomegalovirus infection | 0.448275862 | 1.86227E-12 | CXCL8/SRC/TNF/EGFR/RELA/IL6/MYC/IL1B/CASP3/CCL2/AKT1/PRKACA/TP53 | 13 |
| TNF signaling pathway | 0.379310345 | 2.01406E-12 | IL6/JUN/CSF2/IL1B/CASP3/CCL2/AKT1/CXCL1/TNF/CXCL2/RELA | 11 |
| Rheumatoid arthritis | 0.344827586 | 1.03579E-11 | IL1A/IL6/JUN/CXCL8/CSF2/IL1B/CCL2/CXCL1/TNF/CXCL2 | 10 |
| Kaposi sarcoma-associated herpesvirus infection | 0.413793103 | 1.08246E-11 | IL6/JUN/CXCL8/CSF2/SRC/MYC/CASP3/AKT1/CXCL1/TP53/CXCL2/RELA | 12 |
