# Supplementary material for: Mechanistic insights into triclosan-induced hepatotoxicity: A network toxicology and molecular docking approach
Source: PLoS One. 2026 Feb 25;21(2):e0333244. doi: 10.1371/journal.pone.0333244 (PMC12935200; doi:10.1371/journal.pone.0333244)
Supplement: S6 Table — (DOC) [file pone.0333244.s006.doc]

S5 Table. The top 20 pathways of potential and core targets

| **Group** | **Potential targets (683)** | | | **Core targets (29)** | | |
| --- | --- | --- | --- | --- | --- | --- |
| **Rank** | **Term** | **Count** | **P Value** | **Term** | **Count** | **P Value** |
| 1 | Pathways in cancer | 95 | 1.14E-21 | Lipid and atherosclerosis | 17 | 3.72E-19 |
| 2 | AGE-RAGE signaling pathway in diabetic complications | 39 | 8.03E-21 | IL-17 signaling pathway | 14 | 5.58E-19 |
| 3 | Lipid and atherosclerosis | 55 | 1.35E-19 | AGE-RAGE signaling pathway in diabetic complications | 12 | 6.66E-15 |
| 4 | PI3K-Akt signaling pathway | 68 | 1.16E-16 | Pathways in cancer | 18 | 2.95E-14 |
| 5 | Non-alcoholic fatty liver disease | 42 | 8.62E-16 | Amoebiasis | 11 | 4.61E-13 |
| 6 | Bile secretion | 31 | 5.63E-15 | Chemical carcinogenesis - receptor activation | 13 | 1.03E-12 |
| 7 | HIF-1 signaling pathway | 34 | 7.94E-15 | Human cytomegalovirus infection | 13 | 1.86E-12 |
| 8 | Fluid shear stress and atherosclerosis | 38 | 2.05E-14 | TNF signaling pathway | 11 | 2.01E-12 |
| 9 | Alcoholic liver disease | 38 | 4.22E-14 | Rheumatoid arthritis | 10 | 1.04E-11 |
| 10 | Carbon metabolism | 34 | 4.40E-14 | Kaposi sarcoma-associated herpesvirus infection | 12 | 1.08E-11 |
| 11 | Metabolic pathways | 166 | 1.67E-13 | Fluid shear stress and atherosclerosis | 11 | 1.12E-11 |
| 12 | Apoptosis | 36 | 2.00E-13 | Hepatitis B | 11 | 4.78E-11 |
| 13 | Glycolysis / Gluconeogenesis | 25 | 5.13E-13 | NOD-like receptor signaling pathway | 11 | 2.08E-10 |
| 14 | Endocrine resistance | 30 | 6.96E-13 | Yersinia infection | 10 | 3.10E-10 |
| 15 | TNF signaling pathway | 32 | 3.49E-12 | Legionellosis | 8 | 3.79E-10 |
| 16 | Colorectal cancer | 27 | 6.65E-12 | Proteoglycans in cancer | 11 | 4.41E-10 |
| 17 | IL-17 signaling pathway | 28 | 9.70E-12 | Endocrine resistance | 9 | 6.32E-10 |
| 18 | Hepatitis B | 36 | 5.49E-11 | Non-alcoholic fatty liver disease | 10 | 9.83E-10 |
| 19 | Chemical carcinogenesis - receptor activation | 42 | 6.36E-11 | MAPK signaling pathway | 12 | 1.06E-09 |
| 20 | Adipocytokine signaling pathway | 23 | 9.49E-11 | Inflammatory bowel disease | 8 | 1.24E-09 |
