# Supplementary material for: Mechanistic insights into triclosan-induced hepatotoxicity: A network toxicology and molecular docking approach
Source: PLoS One. 2026 Feb 25;21(2):e0333244. doi: 10.1371/journal.pone.0333244 (PMC12935200; doi:10.1371/journal.pone.0333244)
Supplement: S7 Table — (DOC) [file pone.0333244.s007.doc]

S6 Table. Information sheet of proteins and ligands in molecular docking

| **Protein** | **Structure** | **Released** | **Method** | **Organisms** | **Resolution** | **Ligand** | **Score** |
| --- | --- | --- | --- | --- | --- | --- | --- |
| TP53 | 9fzb | 2025-04-02 | X-RAY DIFFRACTION | Homo sapiens | 1.44A | triclosan | -7.1 |
| EGFR | 8a27 | 2022-10-19 | X-RAY DIFFRACTION | Homo sapiens | 1.07A | triclosan | -7.7 |
| AKT1 | 8r5k | 2024-05-08 | X-RAY DIFFRACTION | Homo sapiens | 0.89A | triclosan | -6.6 |
| IL6 | 7l4v | 2021-11-03 | X-RAY DIFFRACTION | Homo sapiens | 1.75A | triclosan | -5.5 |
| JUN | 9c66 | 2024-08-28 | X-RAY DIFFRACTION | Homo sapiens | 1.40A | triclosan | -6.0 |
| FN1 | 6pol | 2020-05-06 | X-RAY DIFFRACTION | Homo sapiens | 1.8A | triclosan | -6.5 |
| ESR1 | 8c42 | 2023-12-27 | X-RAY DIFFRACTION | Homo sapiens | 1.4A | triclosan | -6.2 |
| TNF | 6q00 | 2020-04-29 | X-RAY DIFFRACTION | Homo sapiens | 0.85A | triclosan | -5.5 |
