# Supplementary material for: Mechanistic insights into triclosan-induced hepatotoxicity: A network toxicology and molecular docking approach
Source: PLoS One. 2026 Feb 25;21(2):e0333244. doi: 10.1371/journal.pone.0333244 (PMC12935200; doi:10.1371/journal.pone.0333244)
Supplement: S8 Table — (DOC) [file pone.0333244.s008.doc]

S8 Table. Gene expression datasets of 8 validated targets

| **Group** | **CTL** | **CTL** | **CTL** | **TRI** | **TRI** | **TRI** |
| --- | --- | --- | --- | --- | --- | --- |
| **Gene ID** | **GSM5176060** | **GSM5176061** | **GSM5176062** | **GSM5176072** | **GSM5176073** | **GSM5176074** |
| TNF | 38.529814 | 42.8581 | 41.918648 | 37.416608 | 36.699708 | 40.21431 |
| AKT1 | 523.178133 | 528.337557 | 539.068096 | 580.99852 | 573.963706 | 582.920217 |
| ESR1 | 96.000151 | 81.998625 | 77.627667 | 77.281721 | 74.1221 | 74.536109 |
| JUN | 6075.276767 | 5931.69929 | 6428.887608 | 7437.261749 | 7629.004208 | 7266.830066 |
| IL6 | 126.217859 | 147.574817 | 143.240333 | 260.327333 | 249.360858 | 214.995902 |
| TP53 | 116.170356 | 121.811445 | 128.79704 | 169.771456 | 169.304153 | 161.530019 |
| FN1 | 813.074817 | 951.818718 | 883.356703 | 697.909343 | 670.780684 | 743.511284 |
| EGFR | 847.171085 | 837.799368 | 829.246194 | 1160.32867 | 1137.540148 | 1105.672622 |
