# Supplementary material for: Mechanistic insights into triclosan-induced hepatotoxicity: A network toxicology and molecular docking approach
Source: PLoS One. 2026 Feb 25;21(2):e0333244. doi: 10.1371/journal.pone.0333244 (PMC12935200; doi:10.1371/journal.pone.0333244)
Supplement: Attachment — Submitted filename: Response to Reviewers.doc [file pone.0333244.s012.doc]

Dear Reviewers and Academic Editor Erica Joy Florentino Macalalad:

Thank you for your letter and for the comments concerning our manuscript entitled "Mechanistic Insights into Triclosan-Induced Hepatotoxicity: A network toxicology and molecular docking approach" (PONE-D-25-48366). Those comments are all valuable and very helpful for revising and improving our paper, as well as the important guiding significance to our research. We have studied the comments carefully and have made corrections which we hope meet with approval. Revised portions are marked in color on the paper. The main corrections in the paper and the responds to the comments are as following:

**Responds to the Editor:**

1. Please include a separate caption for each figure in your manuscript.

**Response:** We've added a title to each figure. **(Please see page 13, line 361)**

2. Please complete all items on the Human Participants Research Checklist that are relevant for your submission, by following this link: http://journals.plos.org/plosone/s/file?id=dc11/PLOSOne_Human_Subjects_Research_Checklist.docx (Contact us at plosone@plos.org if you cannot access the document.) There may be overlap between the checklist items and other queries listed below; please address any duplicated queries both in your response email and on the checklist itself. Upload the completed Human Participants Research Checklist as file type “Other” when you re-submit your manuscript. This document is for internal journal use only and will not be published if your article is accepted. The requested information will help us to assess whether your submission complies with PLOS ONE’s policies and adheres to applicable reporting standards. Note that your manuscript may be rejected if you provide incomplete or inadequate responses to the checklist questions and that changing the ‘Section/Category’ of your article does not affect this requirement.

**Response:** All data in this study came from public databases and did not involve human subjects. We completed Human Participants Research Checklist and uploaded it to the system.

We tried our best to improve the manuscript and made some changes. These changes will not influence the content and framework of the paper. Moreover, here we did not list the changes but marked them in red in the revised paper.

We appreciate your warm work earnestly and hope that the correction will be approved.

Once again, thank you very much for your comments and suggestions.
